# Implication of the Identification of an Earlier Pseudorabies Virus (PRV) Strain HLJ-2013 to the Evolution of Chinese PRVs

**DOI:** 10.3389/fmicb.2020.612474

**Published:** 2020-12-15

**Authors:** Huimin Liu, Zhibin Shi, Chunguo Liu, Pengfei Wang, Ming Wang, Shida Wang, Zaisi Liu, Lili Wei, Zhenzhao Sun, Xijun He, Jingfei Wang

**Affiliations:** State Key Laboratory of Veterinary Biotechnology, Harbin Veterinary Research Institute, Chinese Academy of Agricultural Sciences, Harbin, China

**Keywords:** pseudorabies virus, isolation, evolution, pathogenicity, china

## Abstract

Pseudorabies viruses (PRVs) pose a great threat to the pig industry of many countries around the world. Human infections with PRV have also been reported occasionally in China. Therefore, understanding the epidemiology and evolution of PRVs is of great importance for disease control in the pig populations and humans as well. In this study, we isolated a PRV designated HLJ-2013 from PRV-positive samples that had been collected in Heilongjiang, China, in 2013. The full genome sequence of the virus was determined to be ∼143 kbp in length using high-throughput sequencing. The genomic sequence identities between this isolate and 21 other previous PRV isolates ranged from 92.4% (with Bartha) to 97.3% (with SC). Phylogenetic analysis based on the full-length genome sequences revealed that PRV HLJ-2013 clustered together with all the Chinese strains in one group belonging to Genotype II, but this virus occurred phylogenetically earlier than all the other Chinese PRV strains. Phylogenetic trees based on both protein-coding genes and non-coding regions revealed that HLJ-2013 probably obtained its genome sequences from three origins: a yet unknown parent virus, the European viruses, and the same ancestor of all Chinese PRVs. Recombination analysis showed that HLJ-2013-like virus possibly donated the main framework of the genome of the Chinese PRVs. HLJ-2013 exhibited cytopathic and growth characteristics similar to that of the Chinese PRV strains SC and HeN1, but its pathogenicity in mice was higher than that of SC and lower than that of HeN1. The identification of HLJ-2013 takes us one step closer to understanding the origin of PRVs in China and provides new knowledge about the evolution of PRVs worldwide.

## Introduction

Pseudorabies virus (PRV) is a member of the genus *Varicellovirus* of the *Alphaherpesvirinae* subfamily within the *Herpesviridae* family. This virus is the causative agent of pseudorabies (Burckstummer et al., 2006), also known as Aujeszky’s disease, in pigs. The genome of PRV is double-stranded linear DNA with a length of ∼143 kbp and encodes approximately 72 genes (Pomeranz et al., 2005). Swine is the natural host and reservoir of PRV, and PRV infection in pigs results in reproductive failure in sows, respiratory disorders in growing and fattening pigs, and a fatality rate of up to 100% in piglets (Lee and Wilson, 1979). This virus can also infect other mammals, such as sheep, goats, cats, dogs, cattle, and raccoons, causing high mortality rates (Schmidt et al., 1987; Marcaccini et al., 2008; Cramer et al., 2011; Sozzi et al., 2014).

The first PRV infection in China was reported in cats in 1947 (Zhou and Sun, 1957), and the disease has since spread to almost all major pig production areas of the country (Tong and Chen, 1999). Beginning in the 1990s, attenuated Pseudorabies (PR) vaccines (Bartha-K61 strain) have been widely used in commercial pig farms, with more than 80% of pigs vaccinated. As a result, the disease has been well controlled (Sun et al., 2016). Since late 2011, however, PR outbreaks have been reported from many Bartha-K61-vaccinated commercial pig farms. Subsequent studies indicated that those outbreaks were caused by infections with a group of emerging PRV strains with antigenic variation (Pol et al., 2013; Wu et al., 2013; Luo et al., 2014; Yu et al., 2014; Wang et al., 2017). To date, variant strains have been reported from more than 23 provinces in China.

PRVs have been classified into the following two genotypes according to phylogenetic characteristics: Genotype I contains isolates from Europe and America, while Genotype II contains isolates primarily from China and other Asian countries (Ye C. et al., 2015; Ye et al., 2016; Wu et al., 2017). Both the earlier Chinese strains (Ea, Fa, and SC) and the novel variant strains (HNX, ZJ01, HeN1, JS, TJ, and HN1201) belong to Genotype II. The Chinese PRV variants exhibit unique indels in their genomes when compared with those of the foreign strains, such as a 6-aa insertion (PAGPGG) between residues 574 and 575 in the N-terminal region of the UL5 protein (Ye C. et al., 2015). To date, the complete genome sequences of PRV strains are still limited, and thus our understanding of the evolution of PRVs worldwide is incomplete.

There is growing evidence that PRV has the potential of causing human diseases. The first suspected human infection with PRV was reported in 1914 and a similar event occurred in 1940. In these two events, the patients were all laboratory workers and developed clinical signs of weakness, sore throat, and itching after exposing to PRV-contaminated materials or infected dogs (Skinner et al., 2001). In China, human PRV infections have been continuously reported in recent years. In 2017, PRV genome sequences were detected in the vitreous humor of a woman who worked on a pig farm. This patient got endophthalmitis 3 days after her eyes were exposed directly to the sewage of the farm (Ai et al., 2018). Another case was reported in 2019, in which the patient suffered from encephalitis and PRV gene fragments were detected in the cerebrospinal fluid and vitreous humor using next-generation sequencing (Wang et al., 2019), after which more cases of encephalitis resulting from PRV infection have been reported in China; most of the patients worked as farmers or veterinarians (Yang H. et al., 2019; Yang X. et al., 2019; Fan et al., 2020; Li et al., 2020; Liu et al., 2020; Ou et al., 2020; Wang et al., 2020), suggesting that PRV infection in humans should not be neglected in the future (Wong et al., 2019).

In this study, we report the isolation and identification of a new PRV strain, HLJ-2013. This virus occurred phylogenetically earlier than all the Chinese PRV strains and showed different pathogenicity in mice compared to PRV strains SC and HeN1. Our findings provide novel insights into the evolution of PRVs in China.

## Materials and Methods

### Ethics Statement

The animal experiment with mice was approved by the Animal Care and Use Committee of Harbin Veterinary Research Institute (ID: HSY-IACUC-2019-152), and the experimental operation was carried out in strict accordance with the recommendations in the Guide for the Care and Use of Laboratory Animals of the Ministry of Science and Technology of the People’s Republic of China.

### Virus Isolation and Identification

PRV-positive tonsil and brain tissues were collected from the diseased piglets, which showed clinical signs of an increased temperature above 40°C, stiffness, and salivation before dying on a farm in Heilongjiang Province, China, 2013. The samples were homogenized in PBS, and the suspensions were harvested by centrifugation at 3,000 × *g* at 4°C for 20 min. Next, the suspensions were filtered through a 0.22-μm filter and inoculated into a confluent monolayer of PK-15 (porcine kidney) cells, which were cultured in Dulbecco’s modified Eagle medium (DMEM) (Invitrogen, CA, United States) supplemented with 10% heat-inactivated fetal bovine serum (FBS) (Gibco, NY, United States), 100 μg/ml streptomycin, and 100 IU/ml penicillin at 37°C with 5% CO_2_. The supernatants were collected after the cells exhibited cytopathic effects (CPEs) at the 3rd passage. The presence of PRV was further confirmed by amplifying a 500-bp fragment of the gE gene using PCR (forward primer 5′-TGG CTC TGC GTG CTG TGC TC-3′ and reverse primer 5′-CAT TCG TCA CTT CCG GTT TC-3′) (Luo et al., 2014).

### Transmission Electron Microscopy (TEM) Examination

The PRV particles were examined using negative staining electron microscopy. The supernatants from cell cultures exhibiting CPEs were centrifuged at 2,000 × *g* for 15 min. Then, the sediments were discarded and the samples were centrifuged again at 11,000 × *g* for 15 min to enrich the viral particles. The pellets were resuspended in 100 μl of PBS. Ten micrograms of the specimen was dropped onto a sheet of parafilm and absorbed with a carbon-coated 300-mesh copper grid by placing the grid (carbon-side down) on the drop for 2 min. The excess fluid was wicked away with filter paper. Then, the grid was stained using 2% sodium phosphotungstate (pH 6.5) for 30 s, after which the excess fluid was wicked away. The prepared grid was loaded and examined on a Hitachi H-7650 transmission electron microscope (Hitachi, TKY, Japan) that was operated at a voltage of 80 kV.

### PRV Genome Sequencing

The total genomic DNA of HLJ-2013 was extracted using a DNA extraction kit (QIAGEN, MD, United States) according to the manufacturer’s instructions. One microgram of the isolated viral genomic DNA was submitted to the Shanghai Hanyu Bio-Tech Co., Ltd. for a determination of the full-length genomic sequence using NGS technology and Illumina paired-end sequencing. A total of 8,343,311 raw reads were obtained, and those containing N-base, adapter sequence, and reads less than 50 nt were removed by using the Trimmomatic software (Version 0.32), generating 6,410,874 clean reads for the following analysis. The reads of pig genomes were separated by comparing with the Boar genome using Blast (NCBI). Then, the reads related to PRV genomes were joined into longer contigs using velvet (Version 1.2.03) (Zerbino and Birney, 2008). The final assembly was performed using GapClosing with the reference PRV genome (GenBank NO. NC_006151). The spanned sequences were identified, and the remaining gaps were amplified with the Universal PCR Primer and Index (X) Primer, which were designed according to the assembled and reference PRV genome sequences. The gaps were amplified using LA *Taq* polymerase (with 2 × GC buffer) (TaKaRa, LN, China) and purified using a gel extraction kit (OMEGA, GA, United States). Finally, the purified PCR product was cloned into the pMD19-T vector for sequencing.

### Preparation of Positive Serum for PRV Strain HLJ-2013

The supernatant of PRV HLJ-2013-infected cells was inactivated by incubating with 0.3% formaldehyde overnight. A mixture of the inactivated viral suspension with Freund’s complete adjuvant was injected into mice through an intramuscular route at a dosage of 100 μl. ELISA was performed to detect the antibody level using the serum that was collected from the caudal vein of the mice 2 weeks post-immunization. Anti-HLJ-2013 positive serum was separated from blood clot samples by centrifugation at 3,000 × *g* for 15 min.

### CPEs and Indirect Immunofluorescence Assay (IFA)

Monolayers of PK-15 and Vero cells were inoculated with PRV strains HLJ-2013, SC (Ye et al., 2016), or HeN1 (Ye C. et al., 2015) at a multiplicity of infection (MOI) of 1, after which the cells were examined daily for CPEs. IFA experiment was performed as the previous study (Li et al., 2019). Briefly, PK-15 cells were infected with PRV strain HLJ-2013, SC, or HeN1 (1 MOI). At 20 h post-infection, the cells were fixed for 20 min with 4% paraformaldehyde at room temperature. After the fixation, the cells were incubated in permeabilizing solution (0.1% Triton X-100 in PBS) for 20 min at room temperature and then incubated in blocking solution buffer (1% BSA in PBS) for 1 h at room temperature. The primary antibodies, including anti-HLJ-2013 positive mouse serum and negative mouse serum, diluted with the permeabilizing solution buffer covered the cells for 2 h at room temperature. Then, the cells were stained with the fluorophore-labeled secondary antibody Alexa Fluor 488 goat anti-mouse IgG (1:500, Sigma-Aldrich, CA, United States) at room temperature for 1 h. The stained cells were visualized using an EVOS FL Auto 2 fluorescence microscope.

### Homology and Phylogenetic Analyses

Homology and phylogenetic analyses were performed on the full-length PRV genome sequences of HLJ-2013 and 21 reference strains (Table 1) downloaded from the GenBank database. The multiple sequence alignment was performed with a high-speed and iterative refinement method (FFT-NS-i) implemented in Mafft (Version 7.471) (Katoh and Standley, 2013). A Maximum-Likelihood (ML) Polygenetic tree based on the complete genome sequences of the PRVs was constructed with the HKY85 model in PhyML software (Version 3.1) (Guindon et al., 2009) and was viewed in Figtree (Version 1.4.3). To verify the ML tree, a maximum clade credibility (MCC) tree based on the complete genome sequences of the PRVs was constructed using the BEAST software (Version 1.10.1) with the methods of a GTR + gamma substitution model, the uncorrelated relaxed clock with a gamma distribution, and the GMRF Bayesian Skyride tree prior (Suchard et al., 2018). A Markov Chain Monte Carlo (MCMC) chain was run with 10,000,000 steps and sampled every 1,000 steps. The first 10% of samples were cut off as burn-in by the TreeAnnotator program in the BEAST package. The default value for other parameters is recommended by the BEAST software. The tree was viewed in Figtree (Version 1.4.3).

**TABLE 1:** Information of PRV reference strains used in this study.

Virus name	Accession No.	References
JS-2012	KP257591	Ye C. et al., 2015
ZJ01	KM061380	—
HNX	KM189912	Ye S. et al., 2015
HNB	KM189914	Yu et al., 2016b
JX/CH/2016	MK806387	—
GD0304	MH582511	—
HN1201	KP722022	Xiang et al., 2016
HLJ8	KT824771	Ye et al., 2016
TJ	KJ789182	Luo et al., 2014
SHSV1	KU056477	—
DL14/08	KU360259	—
HeN1	KP098534	Ye C. et al., 2015
Fa	KM189913	Yu et al., 2016a
Ea	KU315430	—
SC	KT809429	Ye et al., 2016
Bartha	JF797217	Szpara et al., 2011
Kolchis	KT983811	Papageorgiou et al., 2016
Kaplan	JF797218	Szpara et al., 2011
NIA3	KU900059	Mathijs et al., 2016
Becker	JF797219	Szpara et al., 2011
ADV32571/Italy2014	KU198433	—

*^∗^: “—” was represented no reference.*

The phylogenetic trees based on the protein-coding genes and the non-coding regions were constructed using the ML algorithm implemented in MEGA (Version 6.06) (Tamura et al., 2013) and evaluated by the bootstrap method (1,000 replicates).

### Recombination Analysis

To explore the potential contributions of HLJ-2013 to the evolution of Chinese PRV strains, the full-length genome sequences of three PRV strains, including HLJ-2013, SC, and Bartha, were aligned using MAFFT (Version 7.471). Recombination analysis was performed using RDP (Version 4.100) (Martin et al., 2015). A recombination event was considered to be reliable when the significance of *p* < 0.01 was showed in at least three of seven selected algorithms: RDP, Chimaera, BootScan, 3Seq, GENECONV, MaxChi, and SiScan (Yu et al., 2020). Recombination events in PRV strain SC were counted and the UPGMA trees of regions derived from the major parent and the minor parent were also constructed. Then, the PRV strain SC served as the query object to further validate the recombination relationship with other parental candidates (HLJ-2013 and Bartha) using Simplot (Version 3.5.1) (Lole et al., 1999). The parameters were set to a sliding window of 3,000 bp and a step size of 200 bp and gap stripped, and the substitution model was named Kimura 2-parameter.

### Divergence Analysis of PRV Proteins

The multiple sequence alignment for the PRV protein sequences was performed in MEGA (Version 6.06). The percentage of amino acids difference between HLJ-2013 and the reference strains was calculated by MegAlign (DNASTAR package) and visualized as a heatmap generated using R software (Version 3.5.2).

### Growth Kinetics in Cell Lines

The growth kinetics of HLJ-2013, SC, and HeN1 were determined by a one-step growth curve as described previously (Luo et al., 2014). Briefly, the confluent monolayer of PK-15 cells in three 12-well cell culture plates (Corning, MA, United States) was inoculated with HLJ-2013, SC, or HeN1 at an MOI of 10. After 1 h of incubation on ice, the inoculation was replaced with prewarmed fresh media, and the cells were incubated at 37°C for 1 h. Then, the cell culture was harvested at 0, 4, 8, 12, 16, 20, 24, 28, 32, 36, and 40 h. After two freeze–thaw cycles, the cellular debris was removed by centrifugation (600 × *g*, 10 min), and the supernatant was titrated onto PK-15 cells. The experiment was repeated three times to obtain the means.

### Animal Experiment

To determine the pathogenicity of the HLJ-2013, a total of 20 42-day-old mice were divided into four groups, three were experimental groups and the other was treated as the control group. Mice in each of the experimental groups were inoculated with 20 μl 10^5^ TCID_50_ of HLJ-2013, HeN1 (Variant strains), or SC (Classical strain) via the intranasal route. The mice in the control group were inoculated with serum-free DMEM of the same dose and route. The mortality of mice was observed daily. After 7-day post-inoculation (dpi), all the surviving mice were euthanized and necropsied.

### Histopathology

Brain tissue samples of the mice were fixed with 10% formalin immediately after necropsied. The HE staining was operated automatically by a Leica fully automatic dyeing machine according to standard procedures.

### Nucleotide Sequence Accession Number

The whole genome sequence of HLJ-2013 has been deposited in the GenBank database under the accession number MK080279.

## Results

### Virus Identification

**FIGURE 1:**
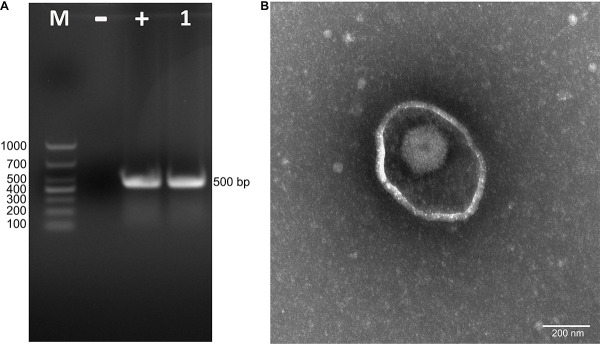
Isolation and identification of PRV strain HLJ-2013. **(A)** PCR assay to detect PRV in PK-15 cells. The cells were inoculated with suspected PRV-containing samples and subjected to PCR amplification using primers specific for a segment of the PRV gE gene. **(B)** Negative staining electron micrograph of PRV particles.

**FIGURE 2:**
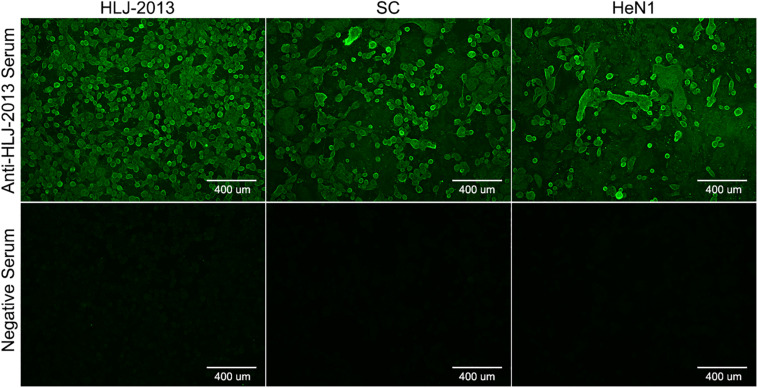
Indirect immunofluorescent assay (IFA) for the detection of PRV strain HLJ-2013. PK-15 cells infected with PRV strain HLJ-2013, SC, and HeN1 strains were incubated with a positive serum of HLJ-2013 and negative serum.

To isolate PRV from positive tissue samples, the PK-15 cells were inoculated with the tissue homogenate. After three times of passaging, the cells showed typical CPEs of PRV infection. The viral DNA was then extracted and amplified using specific primers targeting at a 500-bp segment of the gE of PRV. The PCR results proved that the cell supernatant was positive for the gE fragment (Figure 1A). To further confirm the PCR results, negative staining samples were prepared using the cell supernatant and examined using an EM. Intact herpes viral particles were found in the samples (Figure 1B). Furthermore, to verify the isolation, we prepared the positive serum of the new virus. IFA results showed that strong fluorescent signals were detected in the cells infected with the new virus and PRV strains SC and HeN1 (Figure 2). These results showed that a PRV has been successfully isolated from the tissue samples. The virus was designated as HLJ-2013, according to the place and time of the sample collection.

### Genomic Characteristics and Phylogenetic Analysis of PRV HLJ-2013

**FIGURE 3:**
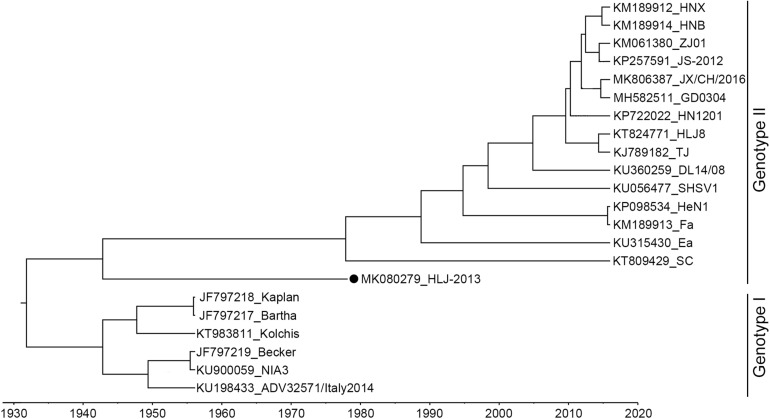
MCC tree of the full-length genome sequence of HLJ-2013 and 21 reference strains. PRVs were identified as two genotypes, Genotype I, and Genotype II. Each reference PRV strain is labeled with its name and the GenBank accession number. PRV strain HLJ-2013, isolated in this study, is labeled with a black dot (•). Transverse axis shows timeline in units of years.

The complete genome of HLJ-2013 was determined using high-throughput sequencing. It was 142.56 kbp in length, with the GC content up to 73.67%. Homology analysis showed that the HLJ-2013 shared genomic sequence identities ranging from 92.4% (with Bartha) to 97.3% (with SC) with the previous PRV isolates. To infer the phylogeny of the virus, an ML tree was initially constructed based on the full-length genome sequences of the virus and 21 reference PRV strains. In the tree, HLJ-2013 and all Chinese isolates were clustered together in one branch belonging to Genotype II. However, HLJ-2013 occurred phylogenetically earlier than all the other Chinese isolates. To further verify the ML tree, we constructed a MCC tree to infer the date of the most recent common ancestor (MRCA) for the PRVs. In the MCC tree, a consistent topological structure was obtained as the ML tree. Both the trees revealed that the PRV strain HLJ-2013 occurred earlier than all the Chinese PRV strains. The time of its occurrence was predicted to be in the 1940s (Figure 3). These results showed that HLJ-2013 is the oldest PRV strain by far and evolved independently from the other Chinese PRV strains.

**FIGURE 4:**
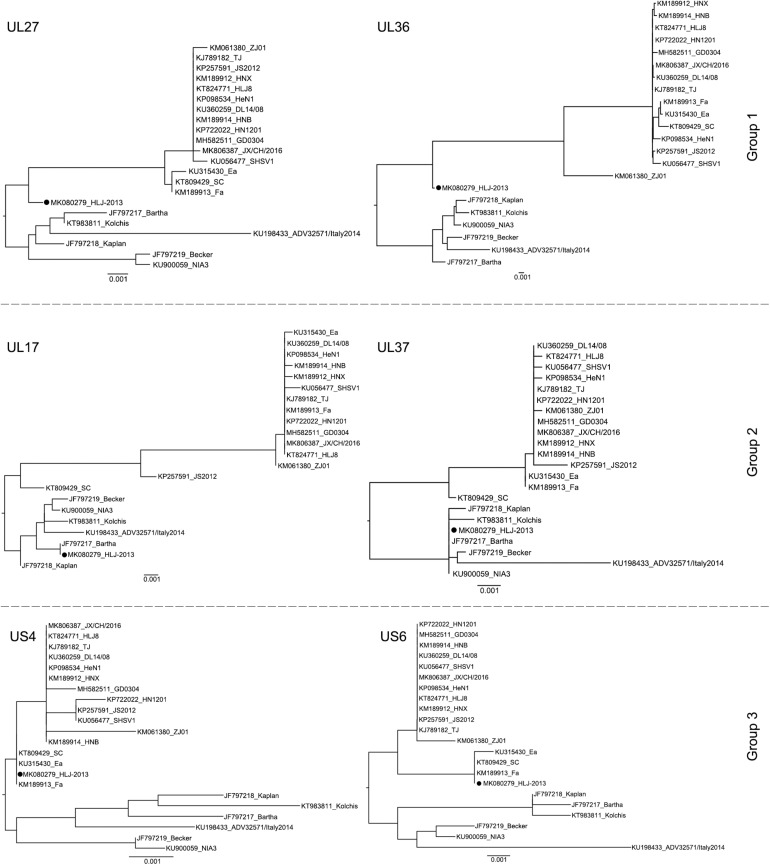
ML trees based on the nucleotide sequences of the UL27 (gB), UL36 (VP1/2), UL17 (pUL17), UL37 (pUL37), US4 (gG), and US6 (gD) genes. All reference strains are labeled with its name and the GenBank accession number. PRV strain HLJ-2013 is marked with a black dot (•).

**FIGURE 5:**
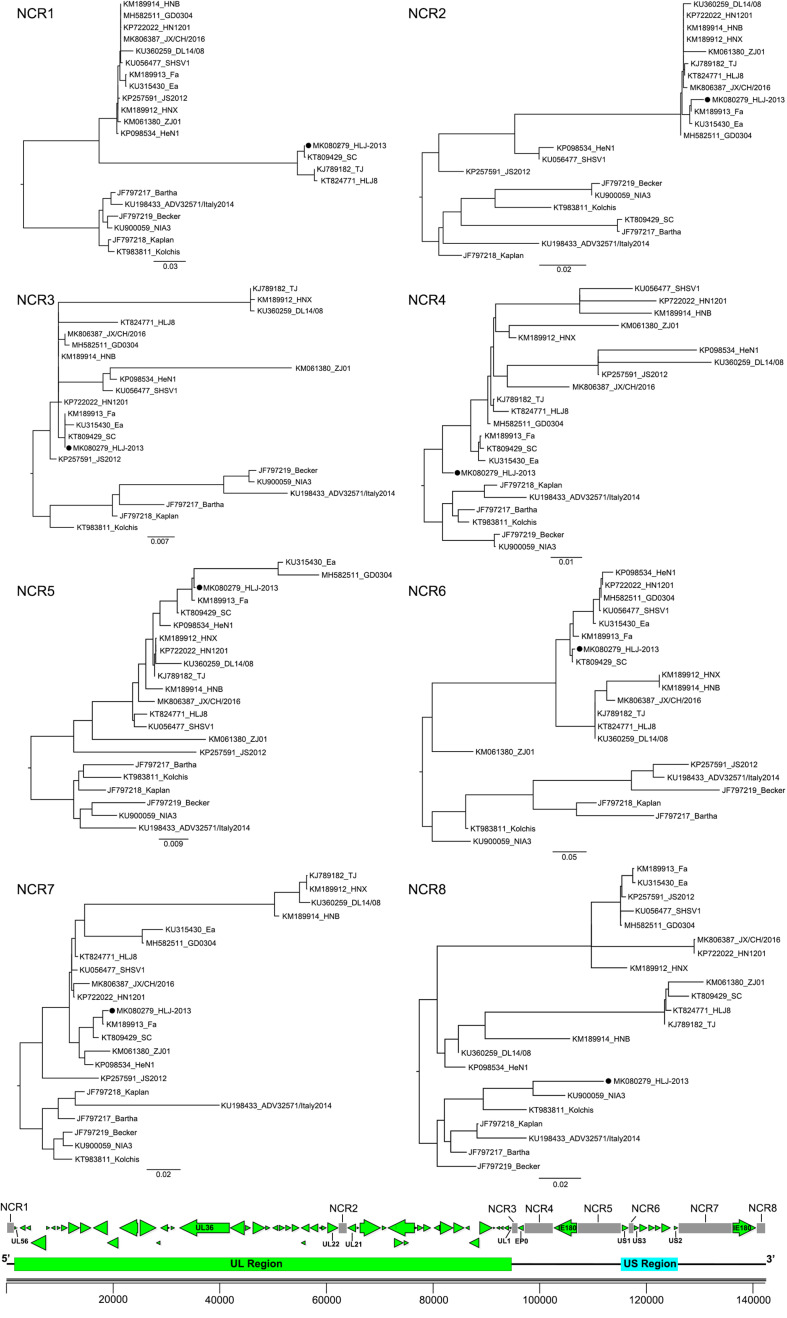
ML trees based on the non-coding regions of the PRV strains. All reference strains are labeled with its name and the GenBank accession number. PRV strain HLJ-2013 is marked with a black dot (•). The position of each non-coding region is shown on the genome frame diagram of PRV.

To further study the evolutionary characteristics of HLJ-2013, phylogenetic trees based on the protein-coding genes and the non-coding regions (NCR) of HLJ-2013 and reference strains were constructed. The phylogenetic trees based on the protein-coding genes of HLJ-2013 could be classified into three groups in terms of the tree’s structural topology. In Group 1, the trees based on the genes, such as UL27 (gB), UL36 (VP1/2), and UL13 (pUL13), showed a similar topological structure to the full-length genome tree, in which those genes were designated in a unique sub-branch and occurred phylogenetically earlier than all the other Chinese isolates. In Group 2, a total of 18 genes of HLJ-2013 (including UL3, UL5, UL6, UL7, UL11, UL15, UL16, UL17, UL23, UL24, UL28, UL37, UL38, UL39, UL40, UL41, UL42, and UL43) showed similar phylogenetic characteristics as represented by the trees of UL17 (pUL17), and UL37 (pUL37); those genes of HLJ-2013 were clustered with the European strains in the same branch. In Group 3, the trees of 39 genes of HLJ-2013 (including EP0, IE180, UL2, UL3.5, UL4, UL9, UL14, UL18, UL19, UL20, UL21, UL22, UL26, UL26.5, UL29, UL31, UL32, UL33, UL34, UL35, UL44, UL46, UL47, UL49, UL49.5, UL50, UL51, UL52, UL53, UL54, UL56, US1, US2, US3, US4, US6, US7, US8, and US9), which were represented by US4 (coding gG) and US6 (coding gD), were clustered with the earlier Chinese PRV strains in one sub-branch (Figure 4). Furthermore, phylogenetic analysis of the non-coding regions showed similar phylogenies with the protein-coding regions. Most of the non-coding regions of HLJ-2013 (NCR1, 2, 3, 5, 6, and 7) shared a close relationship with the earlier Chinese strains. In the phylogenetic tree based on NCR4, HLJ-2013 was designated to a unique sub-branch just like the full-length genome tree. Not surprisingly, HLJ-2013 was clustered in the branch that was composed of the European strains revealed by the tree of NCR8 (Figure 5). Taken together, these phylogenetic analysis results revealed that the genome frame of HLJ-2013 was probably gained from multiple origins.

### Recombination Analysis

**FIGURE 6:**
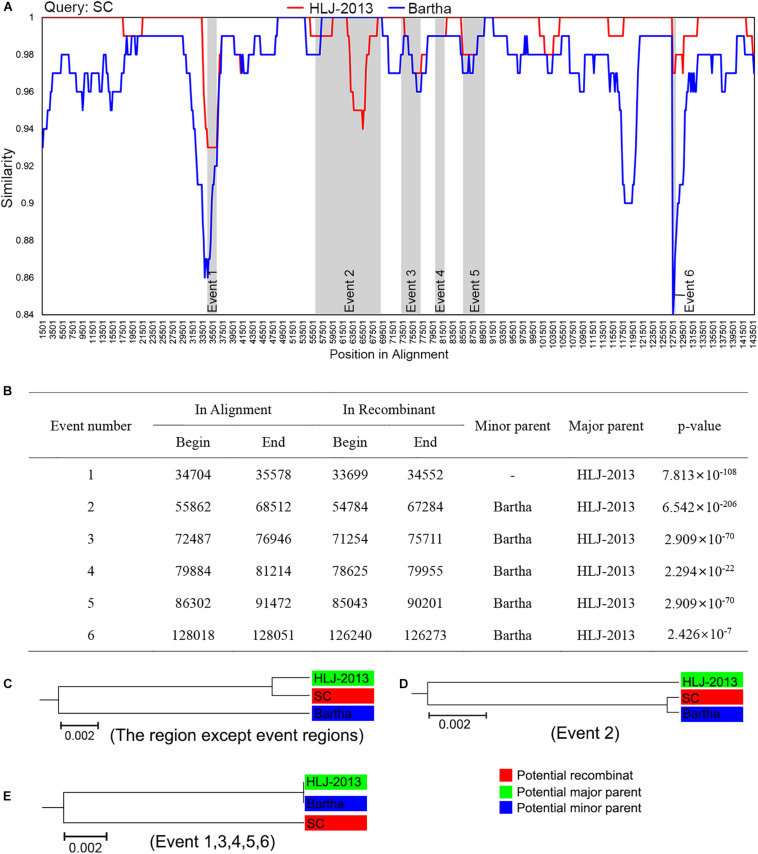
Recombination analysis of PRV strain SC against HLJ-2013 and Bartha. **(A)** Genome-scale similarity comparisons of PRV variant strain SC (query) against strain HLJ-2013 and Bartha. The parameter of the recombination analysis: Window, 3,000 bp; Step, 200 bp; GapStrip, on; Kimura (2-parameter); T/t, 2.0. **(B)** Potential recombination events occurred in PRV strain SC. **(C)** UPGMA tree of regions except occurred recombination event regions derived from the potential major parent and the potential minor parent. **(D)** UPGMA tree of recombination event region 2 derived from the potential major parent and the potential minor parent. **(E)** UPGMA tree of recombination event region 1, 3, 4, 5, and 6 derived from the potential major parent and the potential minor parent.

Given HLJ-2013 was predicted to be probably older than all the other Chinese PRV strains, we explored the potential contributions of HLJ-2013 to the evolution of Chinese PRVs by using a recombination analysis. The earlier PRV strain SC was used as the query object to identify potential recombination events against PRV candidates HLJ-2013 and Bartha. The results showed that six possible recombination events occurred in the genome of SC (Figures 6A,B). In those recombination events, HLJ-2013 was predicted to be the major parent and Bartha was the minor parent of SC. Still, there were several regions in the genome of SC that showed very low similarity with both HLJ-2013 and Bartha, suggesting that there was probably another origin of these fragments. To further analyze these recombination events, UPGMA trees based on the predicted recombination regions were constructed. SC was assigned together with HLJ-2013 in one branch in the tree based on the fragments without the predicted event regions, suggesting that the major framework of the genome of SC was probably donated by HLJ-2013-like viruses (Figure 6C). In the tree constructed by the Event 2 region, SC and Bartha clustered in one branch (Figure 6D), suggesting that this part of the genome of SC was obtained from the vaccine strain Bartha-K61, which is consistent with the conclusion of a previous study (Ye et al., 2016). The phylogenetic tree constructed using the sequences of the event regions 1, 3, 4, 5, and 6 showed that SC was low in terms of similarity with both HLJ-2013 and Bartha (Figure 6E), suggesting that those regions in the genome of SC had origins other than HLJ-2013 and Bartha as well.

### Amino Acid Divergence Between HLJ-2013 and Other PRV Strains

**FIGURE 7:**
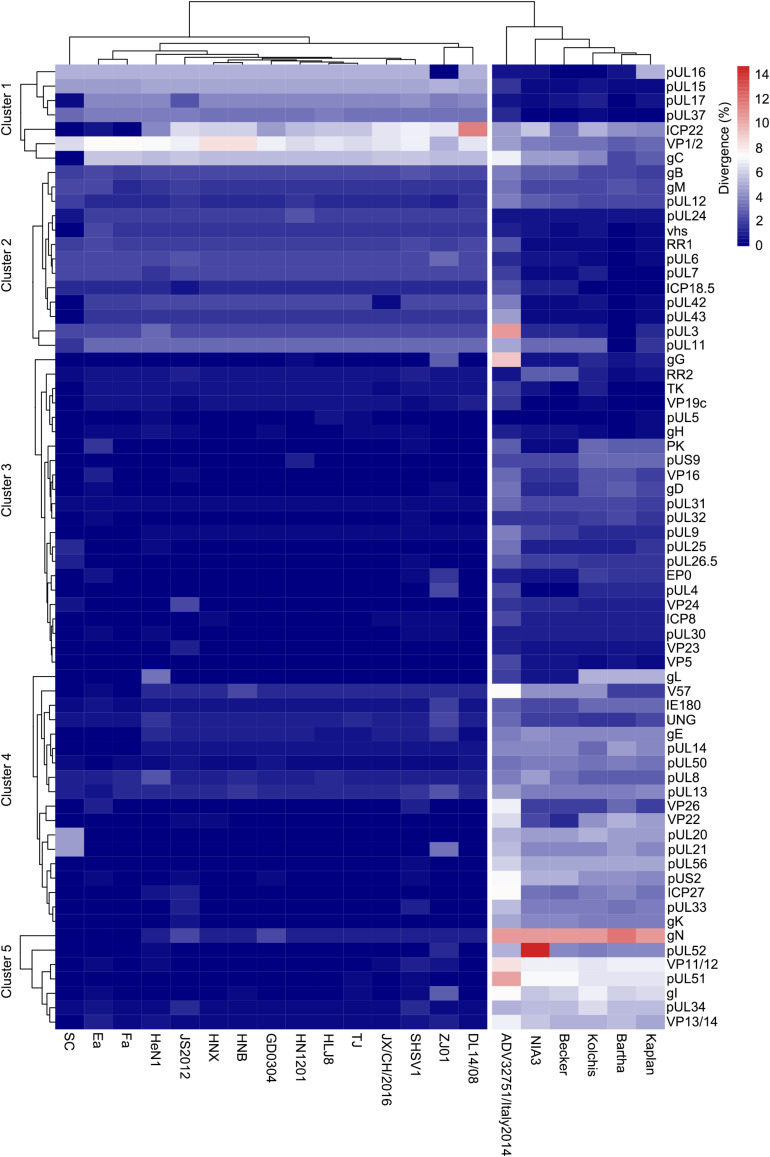
Amino acid divergence between PRV strain HLJ-2013 and the reference strains. The divergency is depicted in colors ranging from dark blue (the lowest) to deep red (the highest).

To further explore the relationship between HLJ-2013 and the reference PRV strains at the amino acid sequence level, we compared the amino acid difference of each protein of HLJ-2013 and 21 other PRVs. A heatmap was generated based on the amino acid divergence of 67 proteins of these viruses (Figure 7). Based on the amino acid divergences, the viruses were classified into two groups. All the Chinese viruses were in one group, while the European strains were designated in another group, suggesting that Chinese PRVs have evolved a unique amino acid distribution pattern that was different from that of the European PRVs. The proteins were classified into five clusters designated Clusters 1–5 as shown in Figure 7. Seven proteins, including the important membrane protein gC, were grouped in Cluster 1. In this cluster, the protein sequence divergences between HLJ-2013 and other Chinese PRVs were the highest compared to the rest of the clusters, but the divergences of the proteins pUL15, pUL16, pUL17, and pUL37 in this group were low between HLJ-2013 and the European viruses, suggesting that these proteins of HLJ-2013 were more similar to the European strains than to the Chinese viruses. Seventeen proteins were in Cluster 2; the protein sequence divergences between HLJ-2013 and all the Chinese viruses were moderate (about 4–6%), but several of which between HLJ-2013 and the European strains, including gB, gM, pUL1, and pUL11, were low, suggesting again that some proteins of HLJ-2013 were probably closer in functions with the European strains. A total of 43 proteins were designated in Clusters 3–5, and all Chinese viruses in these three clusters showed very low divergences, but the European viruses increased gradually, indicating that the majority of biological characteristics of Chinese PRVs were similar.

### Growth Characteristics of PRV HLJ-2013 in Cell Lines

**FIGURE 8:**
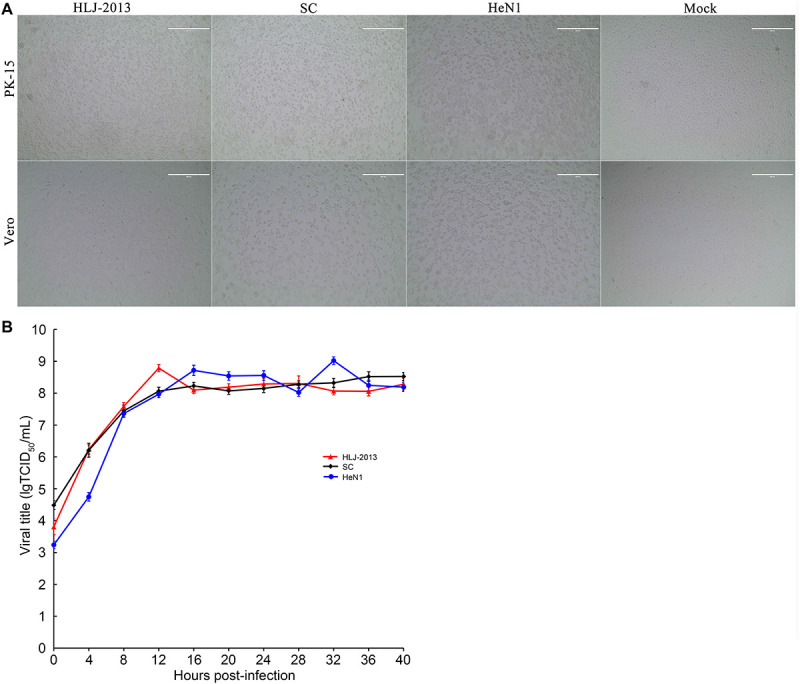
Comparison of cytopathic effects (CPEs) and growth characteristics in infected cells between the PRV strains HLJ-2013, SC, and HeN1. **(A)** CPEs caused by the infections of HLJ-2013, SC, and HeN1 in Vero and PK-15 cells. Vero or PK-15 cells cultured in six-well plates were infected with HLJ-2013 or SC at an MOI of 1 or DMEM as a control. At 24 h post-infection, photographs of CPEs were taken at a magnification of 200×. **(B)** One-step growth curves of the PRV strains. PK-15 cells were infected with HLJ-2013, SC, or HeN1 at an MOI of 10. The cell cultures were harvested at the indicated time points, and titers were determined on PK-15 cells. The mean values with standard deviations of three independent experiments are shown.

To study the replication ability of HLJ-2013 in cell lines, the PK-15 and Vero cells were incubated with PRV strains HLJ-2013, SC, and HeN1. The CPEs caused by HLJ-2013 in both cells were similar to that of SC and HeN1 (Figure 8A). Furthermore, very similar growth kinetics was observed among HLJ-2013, SC, and HeN1 in PK-15 cells (Figure 8B). These findings reveal that HLJ-2013 was similar in terms of cellular growth characteristics with the Chinese classical PRV strain and the PRV variant.

### Pathogenicity of the PRV HLJ-2013 and Histopathologic Examination

**FIGURE 9:**
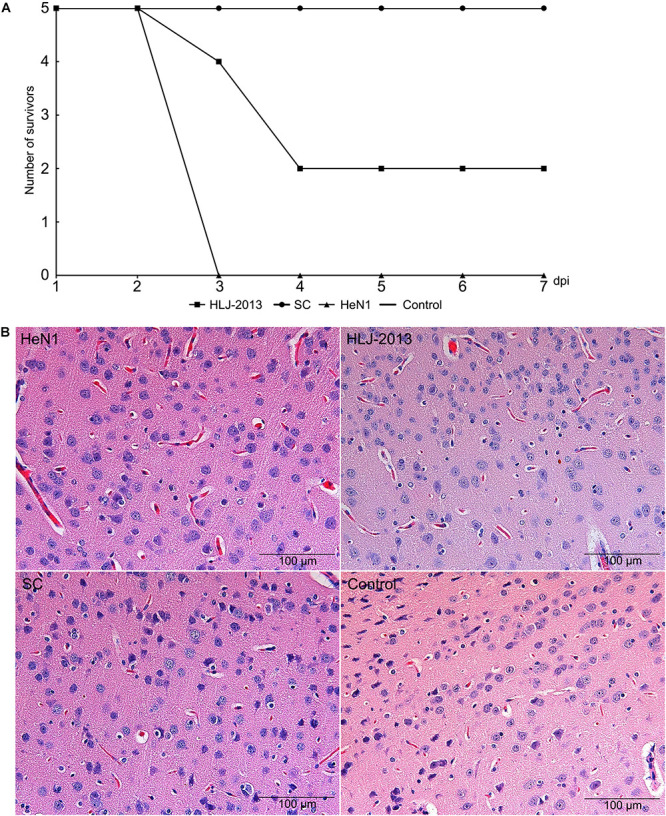
Pathogenicity of different PRV strains in mice. **(A)** The mortality curves of the PRV strains. Mice were inoculated through respiratory tract infection with 20 μl 105 TCID50 of PRV strain, including HLJ-2013, HeN1, and SC. After 7 dpi, the death of each group was calculated. **(B)** Histopathological examination in the brain of the infected and control mice. The pathological changes of brain tissues were observed after infected HLJ-2013, HeN1, and SC strain by HE staining. The microscope scale is 100 μm.

To determine the pathogenicity of the HLJ-2013 in experimental animals, the mice were infected with 20 μl 10^5^ TCID_50_ of PRV strains HLJ-2013, HeN1, or SC through an intranasal route. All the inoculated mice showed obvious clinical symptoms manifested by anorexia, mental fatigue, and neuropathic tic. The mortality rate of PRV strain HLJ-2013, HeN1, and SC was 60% (3/5), 100% (5/5), and zero (0/5), respectively (Figure 9A). Histopathological examinations on the brain tissues of dead mice were performed, and the HE staining showed the proliferation of neuroglial cells and congestion in the brain tissue (Figure 9B). These results manifested that the pathogenicity of HLJ-2013 in mice was weaker than the HeN1 strain, but stronger than the SC strain.

## Discussion

PR is still endemic in the pig populations and poses a continuous threat to the pig industry of China. More importantly, PRV infections in humans have been reported occasionally in recent years (Ai et al., 2018; Yang H. et al., 2019; Fan et al., 2020; Liu et al., 2020). Therefore, fully understanding the evolution and current status of PRVs in China is of great importance to both the pig industry and public health. In this study, we isolated a PRV designated HLJ-2013, and the full-length genome sequence of the virus was determined using the NGS. According to the phylogenetic analysis based on the genome sequences of the virus and reference PRV strains, we found that this virus was an earlier PRV in China. Therefore, it is important to explore the roles it played in the evolution of Chinese PRVs.

Phylogenetic analysis based on the whole genome sequence indicated that HLJ-2013 belonged to the Genotype II PRVs. However, this virus was assigned to a unique sub-branch in the tree, which occurred phylogenetically earlier than all the other Chinese PRV strains. As the probably oldest PRV isolate so far, we were very interested in its origin. Based on the phylogenetic analyses, we found that part of its genome has a unique evolutionary trace indicated by the phylogenetic trees based on the genes such as UL27 and UL36. Another part had a very close relationship with the earlier Chinese PRVs, such as Ea, Fa, and SC (Yu et al., 2016a; Minamiguchi et al., 2019), while the rest of its genome was closer to the European strains, which is consistent with the previous study that indicated that Chinese PRVs obtained part of their genomes from the vaccine strain Bartha-K61 (Ye C. et al., 2015). Together, we believe that PRV strain HLJ-2013 obtained its genome sequence from at least three origins: the first was a uniquely unknown virus; the second and also the majority of the genome framework was the same as the earlier Chinese PRV strains; the third was from the European strains.

HLJ-2013 shared whole genome sequence identities of 92.4–97.3% with 21 other PRV reference strains, the highest of which was with the Chinese classical PRV strain SC, which was also isolated from Heilongjiang Province in the 1980s (Ye et al., 2016). PRV strain SC was believed to be a recombinant of the vaccine strain Bartha-K61 and an unknown parent (Ye et al., 2016). Since HLJ-2013 was probably older even than SC, we then conducted a recombination analysis using SC as a query strain to find potential recombinants in its genome against Bartha-K61 and HLJ-2013. As expected, the major framework of its genome was predicted to have originated from HLJ-2013; the minor parent was Bartha-K61. These results indicate that HLJ-2013-like viruses have played an important role in the evolution of Chinese PRVs.

Viral proteins are responsible for the replication, pathogenicity, and many other functions of the virus. Amino acid divergence presented between a protein of two viruses means that these two viruses may be functioning differently. Through the comparison of the protein divergences between HLJ-2013 and other PRVs, we found that the majority of the proteins of the Chinese PRVs had low divergencies, suggesting that HLJ-2013 probably was similar in most of its biological characteristics with other Chinese PRVs. However, previous studies showed that PRV variants have emerged in the pig populations of China since 2012 and resulted in the failure of vaccination with the vaccine strain Bartha-K61 (An et al., 2013; Wu et al., 2013; Luo et al., 2014; Yu et al., 2014; Gu et al., 2015; Fan et al., 2016; Liu et al., 2017). These variants showed that several insertions existed in the proteins gC, gD, and gE (An et al., 2013; Yu et al., 2014). Our results showed that most of the membrane proteins, including gC, gD, and gE, were moderate in divergences, suggesting that variations existed in these proteins among the Chinese PRV isolates. However, the insertions in the current circulating PRVs were not found in HLJ-2013, suggesting that HLJ-2013 is a classical PRV strain in China. This is consistent with the conclusion obtained from the above phylogenetic analysis based on the full-length genome sequences of HLJ-2013 and reference PRV strains. However, a unique insertion (S^75^P^76^G^77^) was identified in only the gB of HLJ-2013. gB plays an essential role in the membrane fusion and penetration of PRV (Kopp et al., 1994). The functional implication of this insertion of three amino acids in the gB protein of HLJ-2013 needs to be explored in the future.

The phylogenetic and protein difference analyses indicate that HLJ-2013 was possibly different in terms of biological characteristics from the other PRV strains. We then compared the replication in cell lines and pathogenicity in mice among HLJ-2013, classical PRV strain SC, and variant strain HeN1. The susceptible cells (PK15 and Vero cell) infected by HLJ-2013 showed CPEs and growth kinetics similar to those of the earlier Chinese strain SC and the virulent strain HeN1. Mice are susceptible to PRV and infection, which always results in rapid death of almost all the animals. To compare the pathogenicity of HLJ-2013, SC, and HeN1 in mice, we inoculated the mice with a dose of 20 μl 10^5^ TCID_50_ of each virus through the intranasal route. Using the fatality rate as a criterion, the pathogenicity of HLJ-2013 was stronger than that of the SC strain, but weaker than that of the HeN1 strain. In our experiment, mice infected with the SC strain showed pathological changes in brain tissues, but no death was observed, which was different from the previous study that showed a fatality rate of 100% in mice infected with 100 μl 10^5^ TCID_50_ of SC strain through subcutaneous injection (Luo et al., 2014). We suspect that this was possibly caused by different inoculation routes and dosages between these two experiments. Therefore, the pathogenicity of HLJ-2013 in animals, especially in pigs, should be further studied in the future.

Given HLJ-2013 was an earlier PRV strain, we were curious about whether this type of virus is still circulating in the pig populations of China. We then performed an epidemiological survey to detect PRV in pig tonsil samples that were collected by our laboratory in Heilongjiang Province in 2015. A total of 320 samples were detected, but none of them was positive for PRV. Therefore, this question remains unanswered and it should be addressed in subsequent studies.

In this work, we isolated a new PRV strain, HLJ-2013. It is a classical PRV strain and occurred phylogenetically earlier than all the other Chinese PRV isolates. The genome of this virus probably originated from three sources: a yet unknown virus, Genotype I viruses, and the same ancestor of all Chinese PRVs. Recombination analysis suggests that HLJ-2013-like viruses were probably the parents of current circulating PRVs in China. These findings provide new knowledge on the epidemiology and evolution of PRV.

## Data Availability Statement

The full-length genome sequence of PRV strain HLJ-2013 that obtained in this study has been deposited in GenBank database under the accession number of MK080279.

## Ethics Statement

The animal study was reviewed and approved by the Animal Care and Use Committee of Harbin Veterinary Research Institute.

## Author Contributions

JW designed the study. ZL, LW, and ZSu collected the samples. HL and ZSh performed the laboratory work. ZSu and PW carried out the phylogenetic analysis of the complete genome of PRV. CL and MW carried out the phylogenetic analysis of the genes. ZL, LW, and XH provided the experiment support. JW, HL, and ZSh analyzed the data and drafted the manuscript. All authors read and approved the final manuscript.

## Conflict of Interest

The authors declare that the research was conducted in the absence of any commercial or financial relationships that could be construed as a potential conflict of interest.
